# Effects of endovascular recanalization on symptomatic non-acute occlusion of intracranial arteries

**DOI:** 10.1038/s41598-023-31313-4

**Published:** 2023-03-20

**Authors:** Jinchao Xia, Huili Gao, Kun Zhang, Bulang Gao, Tianxiao Li, Ziliang Wang

**Affiliations:** Stroke Center, Henan Provincial People’s Hospital, Zhengzhou University and Henan University, 7 Weiwu Road, Zhengzhou, 450000 Henan China

**Keywords:** Diseases, Medical research, Neurology

## Abstract

To investigate the effect and safety of recanalization surgery for non-acute occlusion of large intracranial arteries and factors affecting clincial outcomes. Patients with non-acute occlusion of internal carotid artery (ICA), middle cerebral artery (MCA), and vertebrobasilar artery (VBA) treated with recanalization were retrospectively enrolled. The clinical and angiographic data were analyzed. 177 patients were enrolled, including 67 patients with intracranial ICA occlusion, 52 with MCA occlusion, and 58 with VBA occlusion. Successful recanalization was achieved in 152 (85.9%) patients. Complications occurred in 15 patients (8.5%). Followed up for 3–7 months, the 90 day mRS was significantly improved compared with that before the procedure. Among 152 patients with successful recanalization, eight patients experienced reocclusion (5.3%), and 11 patients experienced restenosis (7.2%). Successful recanalization was significantly (P < 0.05) associated with occlusion duration, calcification or angulation of the occluded segment. Complications were significantly (P < 0.05) associated with location of occlusion, hyperlipidemia, and patients’ height. Restentosis or reocclusion at follow-up was significantly (P < 0.05) associated with complications and mRS at 90 days. The significant (P < 0.05) independent risk factors were angulation and calcification for successful recanalization, hyperlipidemia for complications, and mRS at 90 days for restenosis or reocclusion at follow-up. Recanalization surgery may be a safe and effective approach for patients with non-acute symptomatic occlusion of large intracranial arteries, and factors significantly independently associated with successful recanalization, periprocedural complications and restenosis or reocclusion after surgery have been identified for future reference to improve clinical outcomes.

## Introduction

Atherosclerotic steno-occlusive disease of large intracranial arteries is a common cause of stroke across the world which is associated with a high risk of recurrent stroke and poor outcomes^1–3^. Non-acute symptomatic atherosclerotic occlusion of large intracranial arteries beyond 24 h from onset is common with a high rate of recurrent stroke of approximately 7.27% per year in China because of hemodynamic compromise and poor collateral circulation and constitutes a treatment dilemma^2,4^. It may affect cognitive function and cause decreased quality of life, thus necessitating timely treatment^5,6^. Patients with acute intracranial large artery occlusion may survive the acute stage but will still experience recurrent transient ischemic attack (TIA) or stroke, with a yearly incidence of 6–14%^7,8^. Some patients can benefit from surgical treatment if they still have recurrent stroke or progressive aggravation of symptoms after intensive drug treatment^9–11^. In earlier studies, low flow extracranial-intracranial artery bypass did not show advantages over drug treatment, nor did it reduce the risk of ipsilateral ischemic stroke^12,13^. Recently, some small-sample studies have been performed on the effect of endovascular recanalization on symptomatic non-acute occlusion of intracranial internal carotid artery (ICA)^14–16^, middle cerebral artery (MCA)^2,17–19^, and vertebrobasilar artery (VBA)^20,21^. For non-acute occlusion of the initial segment of the ICA, hybrid surgery involving endarterectomy and endovascular stenting has achieved a high success rate of 97%^15^, and for non-acute occlusion of intracranial MCA and VBA, endovascular recanalization is the primary approach which may result in heterogeneous outcomes. Currently, the effect and safety of endovascular recanalization for non-acute occlusion of large intracranial arteries are controversial, and significant factors affecting the clinical outcome, periprocedural complications and restenosis or reocclusion after recanalization are yet to be decided. It was hypothesized that endovascular recanalization would be effective and safe for non-acute occlusion of large intracranial arteries of ICA, MCA and VBA. This study was consequently carried out to investigate this hypothesis and significant factors affecting successful recanalization, periprocedural complications, and restenosis or reocclusion after successful recanalization so as to improve the clinical outcome.

## Materials and methods

### Subjects

**Figure 1:**
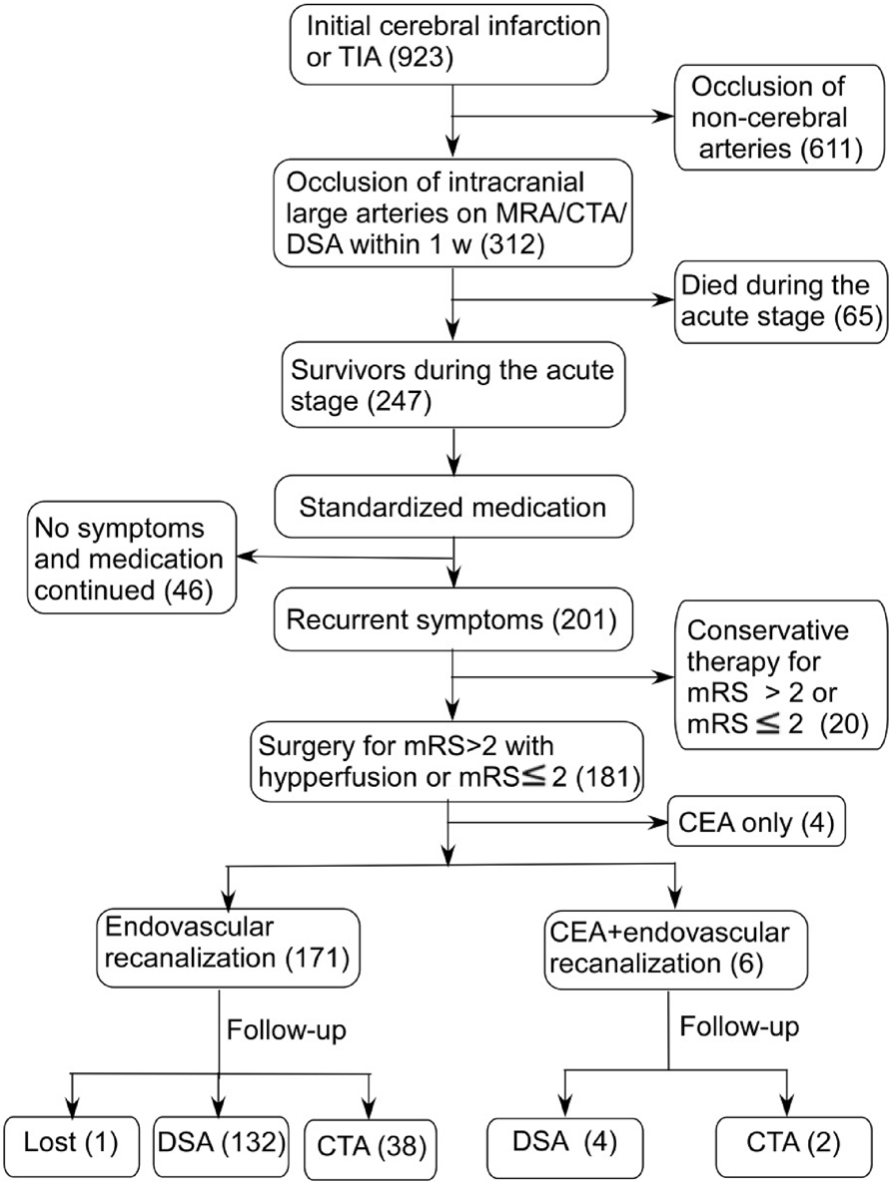
Flow chart of patient enrollment. Twenty patients with mRS > 2 or mRS ≤ 2 were treated with consertive medication because of severe conditions or on requirements of the patients or their family members. CEA, carotid endarterectomy; TIA, transient ischemic attack; MRA, magnetic resonance angiography; CTA, computed tomographic angiography; DSA, digital subtraction angiography; mRS, modified Rankin scale score.

This retrospective case-series one-center study performed in a tertiary hospital was approved by the ethics committee of our hospital, and all patients had provided the written informed consent to participate. All methods were performed in accordance with the relevant guidelines and regulations. Between January 2019 and December 2020, patients with symptomactic non-acute occlusion of intracranial ICA, MCA or VBA treated with endovascular recanalization were enrolled. Non-acute occlusion was defined as occlusion of large intracranial arteries beyond 3 weeks after imaging confirmation of the occlusion. Inclusion criteria were patients with symptomactic non-acute occlusion of intracranial ICA, MCA or VBA confirmed by medical imaging, symptoms of ipsilateral ischemia despite standardized medication, no new ischemic cerebral infarction (within 2 weeks), normal arteries distal to the occlusion without Moyamoya-like vessels, cerebral hemodynamic insufficiency or phase II hemodynamic failure with the mean transit time (MTT) > 4 s, decreased cerebral blood flow (CBF) (symptomatic side/asymptomatic side < 0.95) and increased oxygen extraction fraction (OEF) ratio (ipsilateral/contralateral > 1.13) before endovascular recanalization on computed tomography perfusion (CTP), perfusion weighted imaging (PWI) of magnetic resonance imaging (MRI) or positron emission tomography (PET)^13,22^. Exclusion criteria were patients with severe diseases precluding surgery or anesthesia, heart diseases (unstable angina or acute myocardial infarction), hemorrhagic disease, being allergic or contraindicated to aspirin, clopidogrel, heparin or iodine contrast medium, and elderly patients with advanced diseases or neurological diseases. Standardized medication was applied for initial cerebral infarction. Patients with cerebral infarction were initially treated with standardized medication, including dual antiplatelet therapy and medication for hypertension, hyperlipidemia and diabetes mellitus within 1 month. One month later, single antiplatelet medication and measures for hypertension, hyperlipidemia or diabetes were applied for long-term prevention. If a new infarction occurred within the blood supply area of occluded vessels during medication, it was defined as failure of medical treatment. All patients in this cohort underwent head MRI after occurrence of symptoms to determine the intracranial condition (cerebral infarction, transient ischemic attack or hemorrhage). Then, non-invasive cerebral angiography (MR angiography or CT angiography) and cerebral perfusion examination (MR perfusion or CT perfusion) were performed to determine the patentcy of intracranial vessels and cerebral blood perfusion of the patient. These examinations were completed within 1 week after onset of the disease. If the patient was found to have intracranial arterial occlusion, cerebral digital subtraction angiography was performed to confirm the diagnosis and for possible treatment. The flow chart of enrollment of patients is shown in Fig. 1.

### Pretreatment management

**Figure 2:**
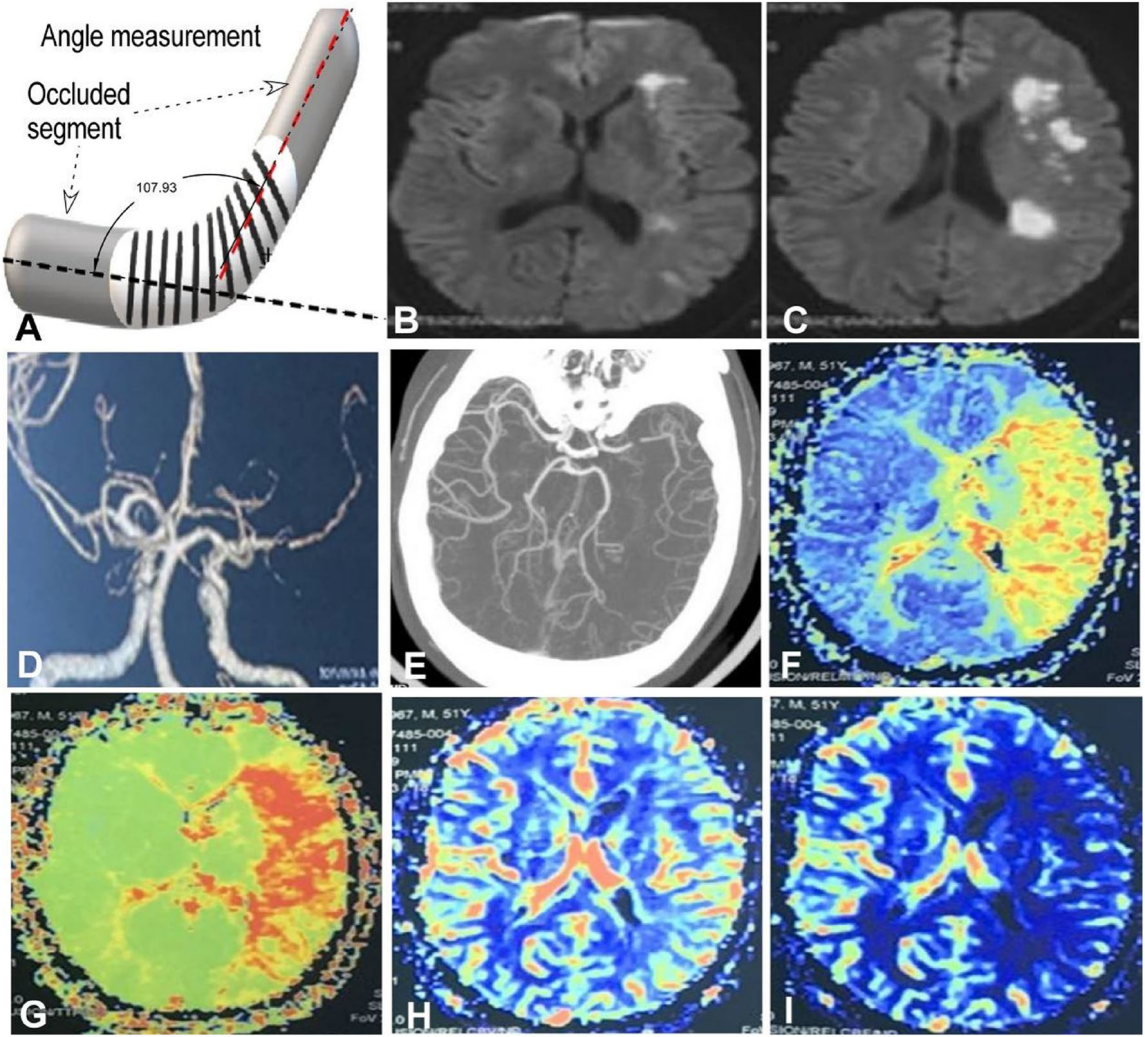
Measurement of angulation at the occluded arterial segment and case presentation. (**A**) The angle formed betwwen the extending line of the proximal segment and that of the distal segment measured 107.93°. (**B**–**I**) A male patient aged 51 years with hypertension for 6 years complained inability to walk, right limb weakness, and unclear speech for 2 days. Physical examination showed the muscle strength of the right limb of grade 2 and normal muscle tension. The modified Rankin scale (mRS) score was 4. (**B**,**C**) Preoperative magnetic resonance imaging (MRI) showed acute cerebral infarction in the left basal ganglia and semioval center. D&E. Cerebral computed tomographic angiography (CTA) revealed occlusion of the M1 segment of left middle cerebral artery. (**F**–**I**) Head MRI perfusion weighted imaging (PWI) confirmed low perfusion in the blood supply area of the left middle cerebral artery. (**F**) Mmean transit time; (**G**) time to peak; (**H**) cerebral blood volume; (**I**) cerebral blood flow.

**Figure 3:**
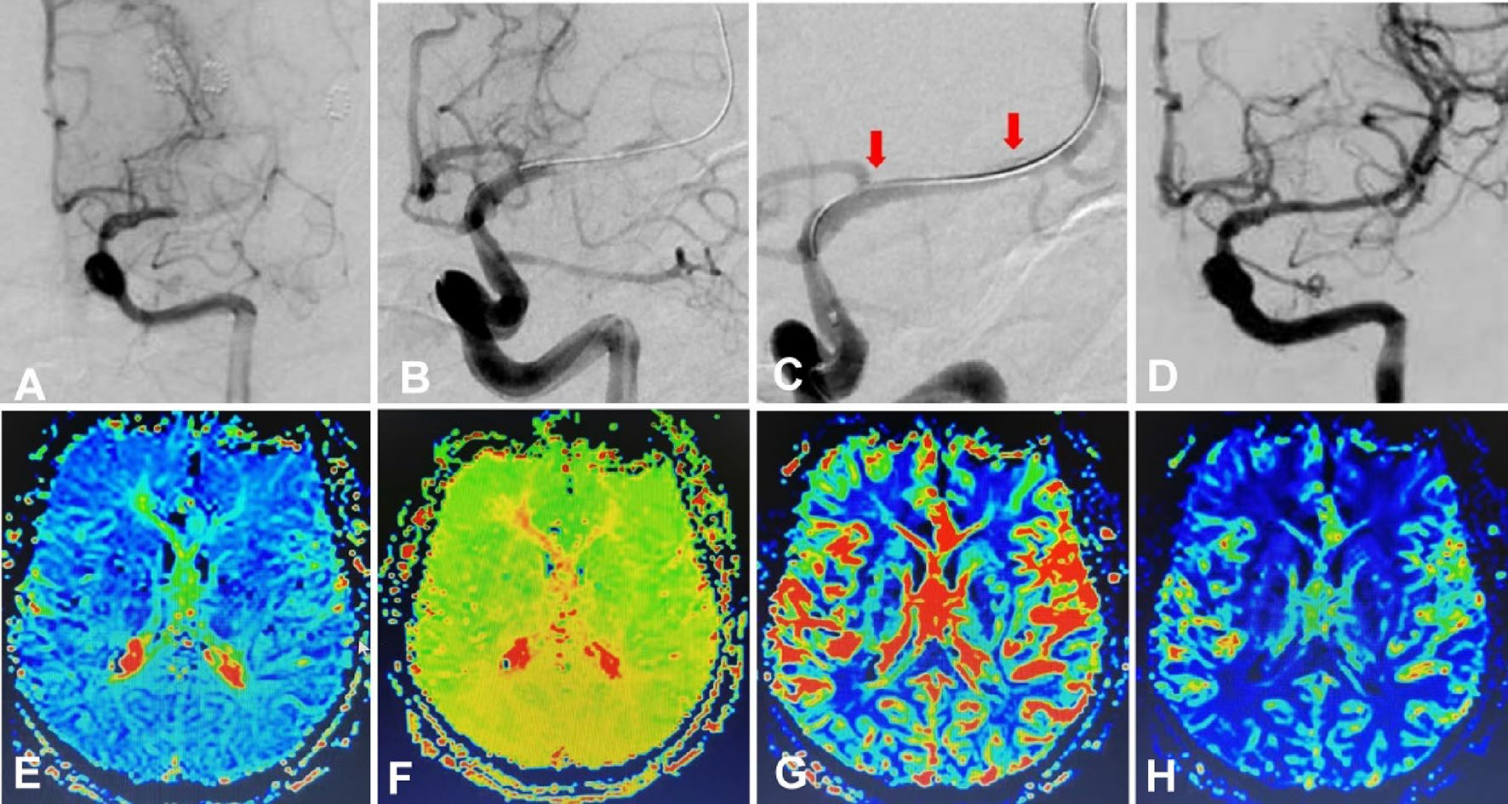
Same patient as in Fig. 2. Endovascular recanalization and magnetic resonance imaging perfusion weighted imaging (MRI PWI). (**A**–**D**) Endovascular treatment was performed 12 days after being hospitalized with a stent being deployed. (**A**) The M1 segment of left middle cerebral artery was occluded on digital subtraction angiography. (**B**) A micro-guidewire was used to explore the occluded segment. (**C**) An Enterprise stent (4.5 * 14 mm, Codman Neuro, New Brunswick, NJ, USA) was deployed at the occluded segment, and two red arrows indicate the markers at the proximal and distal ends of the stent. (**D**) At 6-month follow-up, the stented segment remained patent. (**E**–**H**) MRI PWI 5 days after operation demonstrated that bilateral perfusion was basically symmetrical and left cerebral perfusion was significantly improved. (**E**) Mean transit time; (**F**) Time to peak; (**G**) Cerebral blood volume; (**H**) cerebral blood flow. Immediately after the recanalization procedure, the modified Rankin Scale (mRS) score remained 4. At 4 month follow-up, the mRS was 1.

Digital subtraction angiography and CT angiography images were collected and evaluated by two senior neurointerventional doctors (deputy director or chief physician with experience of over 5 years). The length of occluded vessels, occluded sites, collateral circulation, hypoperfusion variables and neurological symtpoms were collected and analyzed. The measurement of vascular occlusion length was performed using three-dimensional (3D) angiography images from the proximal end to the distal end of occlusion without interference by potential curves of blood vessels. Cerebral hemodynamics and perfusion-diffusion mismatch were evaluated using MRI PWI (Figs. 2 and 3), CTP, or PET. Five days before endovascular recanalization, all patients received dual antiplatelet therapy with aspirin 100 mg/d and clopidorgrel 75 mg/d, and platelet reactivity was evaluated by thromboelastography before the procedure. None of the patients in this group had aspirin resistance. In 22 patients who were resistant to clopidogrel, ticagrelor (90 mg) was used twice daily as a replacement for clopidogrel.

### Endovascular procedure

The endovascular procedure was performed under general anesthesia, and femoral artery puncture was performed for cerebral angiography to observe the patency and anatomical structure of cerebral vessels. Meanwhile, heparin was injected intravenously to maintain the activated coagulation time of 200–250 ms. After an 8F or 6F guiding catheter was inserted into the common carotid artery, ICA or vertebral artery, an Echelon-10 combined 0.014 in. Transend micro-guidewire (Stryker neurovascular, Fremont, CA, USA) or a Pilot micro-guidewire (Abbott vascular, St. Clara, CA, USA) combined with a microcatheter (Medtronic, Minneapolis, Minnesota, USA) was used to explore the occluded blood vessels. If patients with occlusion of the initial part of the internal carotid artery which presented some difficulties in the procedure of endovascular recanalization, a hybrid approach combining both endarterectomy and endovascular intervention was utilized. When the arterial segment distal to the occlusion was reached, a balloon catheter (Boston Scientific, Natick, MA, USA) was used to expand the occluded segment before stent deployment with a balloon- or self-expandable stent. Balloon dilatation was performed if residual stenoses exceeded 50%, and if the residual stenosis did not respond to balloon dilatation after balloon dilatation had been tried for more than 30 min, the operation was terminated as a procedure failure. If the residual stenosis was less than 20% and the antegrade blood flow of cerebral infarction thrombolysis (TICI) grade IIb or above was established, the recanalization surgery was considered successful^23^. If there was no or little blood return or endovascular devices could not be navigated through the occluded segment, the procedure was also defined as a failure.

### Post treatment management and follow-up

CT scan was performed after surgery to exclude intracranial hemorrhage or new ischemic lesions. Blood pressure was controlled at < 140/90 mmHg for 3 days for patients with hypertension or < 120 mmHg for patients without hypertension. Patients with stent deployment took aspirin (100 mg/day), clopidogrel (75 mg/day) and atorvastatin calcium (20 mg/day) tablets for at least 3 months, and then aspirin (100 mg/day) or clopidogrel (75 mg/day) and atorvastatin calcium from 6 months to 1 year based on clinical symptoms and conditions of the treated artery on cerebral angiography. CT angiography, CTP, MRI PWI, or digital subtraction angiography was carried out 3–6 months after surgery for angiographic follow-up. Recurrent ischemic symptoms, vascular reocclusion rate, and modified Rankin scale score (mRS) were recorded during follow-up.

### Parameters analyzed

Age, sex, height, weight, body mass index (BMI, kg/m^2^), clinical symptoms (cerebral infarction and TIA), hypertension (antihypertensive treatment or diastolic pressure > 90 mm Hg or systolic pressure > 140 mmHg), hyperlipidemia (low-density lipoprotein cholesterol > 1 g/L or on lipid-lowering therapy), diabetes mellitus (on oral antidiabetic insulin treatment or fasting serum glucose > 7 mmol/L on two occasions during hospital stay), alcohol abuse (> two alcoholic drinks per day), smoking history, occlusion of the artery, occlusion duration, length, arterial calcification and angulation were recorded and analyzed. Calcification at the occlusion site was defined as any form of calcification found at the occluded arterial segment by head and neck CT angiography. On CT imaging, calcification was hyperdense foci along the artery with a peak density greater than 130 Hounsfield units^24^. Arterial angulation at the occluded segment was defined as the angle formed between the axial extending line of the proximal segment and that of the distal segment (Fig. 2A). If the occluded end was too short to determine the axial direction, the angle formed at the occluded segment was decided by comparing with that on the normal side.

### Statistical analysis

The statistical analysis was performed with the SPSS software (version 25.0, IBM, Chicago, IL, USA). Categorical data were presented as numbers and percentages and tested with the Chi square test, and measurement data were presented as mean ± standard deviation if in normal distribution and tested with the student t test. If in skew distribution, the measurement data were presented as median and interquartile range and tested with the Mann–Whitney U test. Factors significantly affecting successful recanalization, periprocedural complications and restenosis or reocclusion were analyzed using the univariate and multivariate logistic regression analysis. Receiver operating characteristic (ROC) curve analysis was performed for the risk factors for successful recanalization and restenosis or reocclusion. The statistically significant P value was set as < 0.05.

### Ethical approval and consent to participate

This study was approved by the Ethics Committee of Henan Provincial People’s Hospital, and all patients had provided written informed consent to participate.

### Human and animal ethics

Human ethics guidelines were obeyed in doing this study.

## Results

### Subjects

A total of 177 patients were enrolled, including 67 patients with ICA occlusion, 52 with MCA occlusion, and 58 with VBA occlusion, with a female to male ratio of 51/126 and an age range of 38–80 (mean 58.6 ± 9.3) years (Table 1). Cerebral infarction was presented in 160 patients while TIA in 17. No significant (P > 0.05) difference existed in the age, sex component, height, weight, body mass index (BMI), cerebral infarction/TIA, hypertension, hyperlipidemia, diabetes mellitus, alcohol drinking, smoking history, occlusion duration, and occlusion length among three groups of patients. However, a significant (P < 0.05) difference was found in the pretreatment mRS, calcification in the stenosis and angulation of the stenosis (Table 1).

**Table 1:** Demography of patients with occlusoion of large intracranial arteries (range, mean).

Variables	Total (n = 177)	ICA occlusion (n = 67)	MCA occlusion (n = 52)	VBA occlusion (n = 58)	P
F/M	51/126	17/50	20/32	14/44	0.22
Age (y)	38–80 (58.6 ± 9.3)	41–78 (58.4 ± 8.7)	38–80 (57.1 ± 10.3)	39–79 (59.9 ± 9.2)	0.34
Height (cm)	150–184 (167.6 ± 7.1)	152–180 (167.5 ± 7.0)	150–184 (167.2 ± 8.2)	156–180 (168.1 ± 6.2)	0.79
Weight (kg)	52–95 (72.7 ± 9.6)	52–91 (71.9 ± 9.2)	52–95 (72.5 ± 10.3)	52–92 (73.8 ± 9.5)	0.54
BMI	19.5–39.3 (26.0 ± 3.2)	19.5–33.2 (25.6 ± 2.8)	20.3–39.3 (26.3 ± 3.9)	20.1–33 (26.1 ± 2.9)	0.51
Infarction/TIA	160/17	58/9	48/4	54/4	0.22
Hypertension	111 (62.7%)	37 (55.2%)	33 (63.5%)	41 (70.1%)	0.18
Diabetes mellitus	69 (39.0%)	24 (35.8%)	22 (42.3%)	23 (39.7%)	0.60
Hyperlipidemia	44 (24.9%)	7 (10.4%)	19 (36.5%)	18 (31.0%)	< 0.01
Alcohol drinking	70 (39.5%)	30 (44.8%)	19 (36.5%)	21 (36.2%)	0.62
Smoking	86 (48.6%)	32 (47.8%)	24 (46.2%)	30 (51.7%)	0.83
Presurgery mRS: 0	84 (47.5%)	43 (64.2%)	21 (40.4%)	20 (34.5%)	< 0.01
1	57 (32.2%)	18 (26.9%)	19 (36.6%)	20 (34.5%)	
2	20 (11.3%)	6 (9.0%)	5 (9.6%)	9 (15.5%)	
3	6 (3.4%)	0	2 (3.8%)	4 (6.9%)	
4	10 (5.7%)	0	5 (9.6%)	5 (8.6%)	
Occlusion duration (d)	12–378 (28, 21.5–41.5)	14–244 (32, 25–68)	12–378 (26, 21.3–41.3)	13–207 (26, 19.8–37.5)	0.055
Occlusion length (mm)	1.9–187.3 (14.6, 5.8–37.4)	3.8–187.3 (58.3, 12.8–92.4)	1.9–14.2 (4.3, 3.2–6.2)	4.4–46.1 (18.9, 13.7–31.5)	0.055
Calcification(n)	47 (26.6%)	23 (34.3%)	5 (9.6%)	19 (32.8%)	0.001
Angulation (º)	0–123.2 (16, 9.1–38.3)	0–123.2 (55.3, 7.2–78.2)	0–32.8 (11.3, 7.3–14.9)	5.8–73.6 (19.5, 14.6–26.1)	< 0.0001

BMI, body mass index; ICA, internal carotid artery; MCA, middle cerebral artery; VBA, vertebrobasilar artery; TIA, transient ischemic attack; mRS, modified Rankim scale score.

Endovascular recanalization among 67 (37.9%) patients with ICA occlusion, six (9.0%) patients underwent carotid endarterectomy of ICA prxomal orifice occlusion and additional stent deployment for recanalization of intracranial occlusion (Table 2). Sixty-one (91.0%) patients experienced endovascular recanalization using stent angioplasty after balloon dilatation, however, nine (13.4%) patients were not recanalized. Self-expandable stents were deployed in 28 (41.8%) patients while balloon-expandable stents in 33 (49.3%). Among 52 patients with MCA occlusion, eight (15.3%) patients underwent balloon angioplasty only, and 38 (73.1%) were treated with deployment of self-expandable stents (Figs. 2 and 3). Recanalization was failed in six (11.5%) patients. Among 58 patients with VBA occlusion, six (10.3%) patients underwent balloon angioplasty alone, and 52 (89.7%) were treated with stent angioplasty, with deployment of self-expandable stents in 29 (50%) patients, balloon-expandable stents in six (10.3%), and both balloon- and self-expandable stents in seven (12.1%). In ten (17.2%) patients, the recanalization procedure failed. Among all patients, 152 (85.9%) patients were successfully recanalized, whereas 25 (14.1%) patients failed. Successful recanalization was achieved in 58 (86.6% or 58/67) patients with ICA occlusion, in 46 (88.5% or 46/52) with MCA occlusion, and in 48 (82.8% or 48/58) with vertebrobasilar artery occlusion, without significant (P > 0.05) differences in the recanalization rate among three groups. A significant (P < 0.0001) difference was detected in the approach for recanalization among three groups (Table 2). After recanalization, the TICI for evaluation of the antegrade blood flow was IIb in 14 (24.1% or 14/58) patients and III in 44 (75.9%) with ICA occlusion, IIb in 19 (41.3%) patients and III in 27 (58.7%) with MCA occlusion, and IIb in 21 (43.8%) patients and III in 27 (56.2%) with VA occlusion. No significant (P > 0.05) difference was found in the score of TICI among three groups.

**Table 2:** Treatment, outcome and follow-up.

Variables	ICA occlusion (n = 67)	MCA occlusion (n = 52)	VA occlusion (n = 58)	Total (n = 177)	P
Treatment methods					< 0.0001
CEA + stenting	6 (9.0%)	0	0	6 (3.4%)	
Balloon dilation only	0	8 (15.4%)	6 (10.3%)	14 (7.9%)	
SE stent	28 (41.8%)	38 (73.1%)	29 (50%)	95(53.7%)	
BE stent	33 (49.3%)	0	6 (10.3%)	39(22.0%)	
BE + SE stent	0	0	7 (12.1%)	7 (4.0%)	
Recanalization rate	86.6% (58/67)	88.5% (46/52)	82.8% (48/58)	85.9% (152/177)	0.65
TICI grade: IIb/III	14/44	19/27	21/27	52/98	0.10
Complication rate	3/67(4.5%)	9/52 (17.3%)	3/58 (5.2%)	15/177 (8.5%)	0.03
Intracranial bleeding	1 (1.5%)	4 (7/7%)	1 (1.7%)	6 (3.4%)	
Brain infarction	2 (3.0%)	4 (7.7%)	2 (3.4%)	8 (4.5%)	
Cardiac arrest	0	1 (1.9%)	0	1 (0.6%)	
CCF	1 (1.5%)	0	0	1 (0.6%)	
Follow-up (m)	3–7 (3, 3–6)	3–6 (3, 3–6)	3–7 (4.2 ± 1.4)	3–7 (5, 3–6)	0.05
90 d mRS					< 0.01
0	52 (77.6%)	37 (71.2%)	55 (94.8%)	144 (81.4%)	
1	10 (14.9%)	11 (21.2%)	0	21 (11.9%)	
2	2 (3.0%)	1 (1.9%)	0	3 (1.7%)	
3	2 (3.0%)	0	1 (1.7%)	3 (1.7%)	
4	1 (1.5%)	1 (1.9%)	2 (3.4%)	4 (2.3%)	
5	0	1 (1.9%)	0	1 (0.6%)	
Restenosis	3 (5.2%)	2 (4.3%)	6 (12.5%)	11 (7.2%)	0.50
Reocclusion	4 (6.9%)	2 (4.3%)	2 (4.2%)	8 (5.3%)	0.67

ICA, internal carotid artery; MCA, middle cerebral artery; VA, vertebral artery; CEA, carotid endarterectomy; SE, self-expandable; BE, balloon-expandable; TICI, cerebral infarction thrombolysis; CCF, carotid cavernous fistula; mRS, modified Rankim scale score.

### Periprocedural complications

In recanalizing ICA occlusion, three (4.5%) patients experienced periprocedural complications within 7 days after the procedure, including one (1.5%) case with cerebral infarction, one (1.5%) with cerebral hemorrhage, and one (1.5%) with internal carotid cavernous fistula. In patients with MCA occlusion, periprocedural complications occurred in five (9.6%) cases within 7 days, with cerebral infarction in two (3.8%) patients, cerebral hemorrhage in one (1.9%), subarachnoid hemorrhage in one (1.9%), and cardiac arrest in one (1.9%). In recanalizing VBA occlusion, three (5.2%) patients experienced complications within 7 days after the procedure, including cerebral infarction in two (3.5%) patients and cerebral hemorrhage in one (1.7%). Among 25 patients who failed the recanalization procedure, four (16%) patients with MCA occlusion experienced complications within 7 days after the procedure, including cerebral infarction in two patients and subarachnoid hemorrhage in the other two. The total complication rate among all patients was 8.5% (15/177), with a complication rate of 4.5% in patients with ICA occlusion, 17.3% in MCA occlusion, and 5.2% in VBA occlusion, with the complication rate in recanlization of MCA occlusion significantly (P < 0.05) greater than that in the other two groups (Table 2).

### Follow-up

Follow-up was performed 3–7 (3, 3–6) months after the procedure (Table 2), and the 90 day mRS was 0 in 144 (81.4%) patients, 1 in 21 (11.9%), 2 in three (1.7%), 3 in three (1.7%), 4 in four (2.3%), and 5 in one (0.6%), significantly (P < 0.0001) improved compared with those before treatment. Among 58 patients with successful recanalization of ICA occlusion, four (6.9%) patients experienced reocclusion, and three (5.2%) exhibited asymptomatic instent restenosis. Cerebral infarction occurred in two patients with reocclusion. Among 46 patients with successful recanalization of MCA occlusion, reocclusion took place in two (4.3%) patients, leading to cerebral infarction, and asymptomatic instent restenosis occurred in two (4.3%) patients. Among 48 patients with successful recanalization of VBA occlusion, reocclusion occurred in two (4.2%) patients, and instent restenosis happened in six (12.5%). Cerebral infarction occurred in two patients with reocclusion and in two patients with instent restenosis. Thus, the total reocclusion rate and instent restenosis rate were 5.1% (8/56) and 7.1%, respectively, among 156 patients with successful recanalization. No significant (P = 0.26) difference existed in the reocclusion or restenosis rate among patients with ICA, MCA and VA occlusion.

Among 25 (13.8%) patients without successful recanalization who were treated with antiplatelet therapy, the mRS score was aggravated in six (24.0%) patients including two patients with ICA occlusion, one with MCA occlusion, and three with vertebrobasilar artery occlusion.

### Factors associated with successful recanalization

Among all patients, successful recanalization was significantly associated with occlusion duration (P = 0.039), calcification (P < 0.0001) or angulation (P = 0.0002) of the occluded segment, with longer occlusion duration, calcification and greater angulation of the occluded segment significantly decreasing the recanalization rate (Table 3). Multivariate logistic regression analysis showed only angulation and calcification significantly (P < 0.01) associated with successful recanalization. For ICA occlusion, univariate regression analysis demonstrated that the successful recanalization rate was significantly (P < 0.05) decreased by occlusion of more segments of ICA (P = 0.02), more calcification (P = 0.0005), and greater angulation of the stenosis segment (P = 0.01), whereas multivariate logistic regression analysis showed only angulation and calcification were significant (P < 0.05) independent factors associated with successful recanalization (Table 3). In recanalization of MCA occlusion, right side occlusion (P = 0.02), occlusion length (P < 0.0001), calcification (P < 0.0001), angulation (P < 0.0001), and occlusion duration (P = 0.04) significantly decreased the recanalization rate in univariate logistic analysis, whereas right side occlusion, occlusion length and angulation were significant (P < 0.05) independent factors for successful recanalization in multivariate logistic analysis (Table 3). In recanalization of VBA occlusion, both univariate and multivariate logistic regression analyses revealed that occlusion length, calcification, angulation, and increased age were significant (P < 0.05) factors associated with successful recanalization (Table 3). ROC curve analysis of continuous variables among the risk factors showed that the cutoff valuae and area under the ROC curve (AUC) of angulation were 76.9° and 0.76, respectively, for ICA recanalization (sensitivity 0.79 and specificity 0.67), 16.0° and 0.99, respectively, for MCA recanalization (sensitivity 0.93 and specificity 1.00), and 29.4° and 0.91, respectively, for VBA recanalization (sensitivity 0.94 and specificity 0.90). The cutoff value and AUC of occlusion length were 7.3 mm and 0.95, respectively, for MCA recanalization (sensitivity 0.93 and specificity 0.83) and 25.2 mm and 0.69, respectively, for VBA recanalization (sensitivity 0.48 and specificity 0.90) (Table 4).

**Table 3:** Factors significantly affecting successful recanalization, complications, restenosis or reocclusion.

Location	Factors	Univariate logistic regression		Multivariate logistic regression	
		χ^2^	P	χ^2^	P
Successful recanalization					
Total patients	Occlusion duration	4.2	0.039		
	Angulation	13.8	0.0002	7.6	0.0059
	Calcification	37.7	< 0.0001	30.9	< 0.0001
ICA	Occlusion of more segments	15.7	0.03		
	Angulation	5.6	0.018	5.9	0.015
	Calcification	13.6	0.0002	14.0	0.0002
MCA	Right side occlusion	5.8	0.02	5.3	0.022
	Occlusion length	21.1	< 0.0001	11.7	0.0006
	Occlusion duration	4.2	0.04		
	Angulation	28.3	< 0.0001	7.8	0.005
	Calcification	15.6	< 0.0001		
VBA		3.9	0.049	13.3	0.0003
	Angulation	27.2	< 0.0001	28.7	< 0.0001
	Calcification	11.7	0.0006	11.4	0.0007
	Age	4.4	0.035	20.4	< 0.0001
Complications	Occlusion location	7.4	0.02		
	Hyperlipidemia	17.3	< 0.0001	7.9	0.002
	Patients’ height	6.1	0.01		
Restenosis or reocclusion	Complications	9.2	0.002		
	Angulation	14.1	0.0002	6.4	0.01

ICA, internal carotid artery; MCA, middle cerebral artery; VBA, vertebrobasilar artery.

**Table 4:** ROC curve analysis of continuous risk factors.

Variables		Cutoff value	Sensitivity	Specificity	Youden index	AUC
ICA recanalization	Angulation	76.9°	0.79	0.67	0.46	0.76
MCA recanalization	Angulation	16.0°	0.93	1.00	0.93	0.99
	Occlusion length	7.3 mm	0.93	0.83	0.77	0.95
VBA recanalization	Angulation	29.4°	0.94	0.90	0.84	0.91
	Occlusion length	25.2 mm	0.48	0.90	0.38	0.69
Restenosis/reocclusion	mRS at 90 days	3	0.26	1.00	0.26	0.59

ROC, receiver operating characteristic; AUC, area under the ROC curve; ICA, internal carotid artery; MCA, middle cerebral artery; VBA, vertebrobasilar artery.

### Factors associated with complications, restenosis or reocclusion

Occurrence of complications was significantly (P < 0.05) associated with location of occlusion, hyperlipidemia, and patients’ height in univariate logistic regression analysis, but only independently significantly (P = 0.002) associated with hyperlipidemia (OR = 7.9, 95% CI 2.1–30.2) in multivariate logistic analysis (Table 3). Hyperlipidemia was significantly (P < 0.05) associated with more complications, with more complications in recanalization of MCA occlusion followed by VBA and ICA.

Occurrence of restentosis or reocclusion was significantly (P < 0.05) associated with complications and mRS at 90 days in univariate logistic regression analyses (Table 3). Multivariate logistic regression analysis revealed only mRS at 90 days as the only independent risk factor for restenosis or reocclusion (Table 3). More serious periprocedural complications and worse mRS at 90 days resulted in a significantly increased rate of restenosis or occlusion, with the presence of restenosis or reocclusion significantly (P < 0.05) more at the ICA ophthalmic segment followed by ICA cavernous segment, VBA, and MCA.

### A scale for successful recanalization of intracranial arteries

Based on the above outcomes, our embolization experience, and studies in the literature^9–11,15,18,22,25–34^, a scoring system for successful recanalization of intracranial arterial occlusion was estalibshed (Table 5). The recanalization risk was classified into three grades: 0–2 scores for a low risk, 3–5 for a medium risk, and 6–8 for a high risk. For patients with a low or medium risk, recanalization is possible and can be performed, whereas for patients with a high risk, recanalization should be carefully balanced between the risk and benefit and may be abandoned if necessary.

**Table 5:** Scores for successful recanalization of non-acute symptomatic occlusion of cerebral arteries.

Variables		Score
Location or length of occlusion (ICA/MCA/VBA)	Cervical ICA, ≤ 5 mm in MCA or V4 segment to middle and lower BA	0
	Cervical-Cavernous ICA, 5–10 mm in MCA or middle and upper BA	1
	Cavernous anterior curve-PCom, ≥ 10 mm in MCA or V3 involved	2
Occlusion duration	< 3 m	0
	> 3 m	1
Calcified occlusion	No	0
	Yes	1
Occlusion nature	Atherosclerotic	0
	Others^a^	1
Occlusion segment	Straight	0
	Angulation ≤ 30°	1
	Angulation > 30°	2
Experience of operators	Over 5 year or 10 patients treated	0
	Inexperienced	1
Total score	Low risk	0–2
	Medium risk	3–5
	High risk	6–8

ICA, internal carotid artery; MCA, middle cerebral artery; VBA, vertebrobasilar artery; BA, basilar artery; PCom, posterior communicating artery.

Others^a^ indicate occlusion caused by inflammation, dissection, radioactive vascular occlusion, or muscular fiber dysplasia.

## Discussion

### Major findings

This study found that recanalization surgery may be a safe and effective approach for patients with non-acute symptomatic occlusion of large intracranial arteries, and factors significantly independently associated with successful recanalization, periprocedural complications and restenosis or reocclusion after surgery have been identified for future reference. Successful ICA recanalization was significantly (P < 0.05) associated with calcification or angulation of the occluded segment, MCA recanalization with angulation, occlusion length and right side occlusion, and VBA recanalization with occlusion length, angulation, calcification and age for VBA occlusion. The significant (P < 0.05) independent risk factors were angulation and calcification for successful recanalization, hyperlipidemia for complications, and mRS at 90 days for restenosis or reocclusion at follow-up.

### Recanalization of ICA occlusion

Anatomically, from the opening to the bifurcation of the ICA, the ICA is relatively long and divided into 7 segments according to the Bouthillier classification approach^35^. Clinically, atherosclerotic stenosis and occlusion of the ICA are common, with frequent local calcification. Success recanalization of ICA occlusion has been reported to range 70–90% with a periprocedural complication rate of 10–20%^25,30,33^. In our study, the successful recanalization rate of ICA occlusion was 87.3% with a periprocedural complication rate of 6.5%. The relatively high successful recanalization rate of ICA occlusion with a low complication rate results firstly from the straightness of the occluded segment, and if the ICA opening is involved, hybrid surgery can be performed using both endarterectomy and endovascular recanalization. The ICA has fewer branching arteries, and dissection caused by a micro-guidewire may not result in severe consequences. At the ICA intracranial clinoid segment, because the anterior choroidal artery and posterior communicating artery are consistent with the axial direction of ICA, a microcatheter or micro-guide wire may easily enter these arteries and cause arterial occlusion or bleeding if operated carelessly and roughly. To prevent possible complications, angiography with multiple projections for monitoring and timely reshaping of the tip of the micro-guide wire are the key. The superior pituitary artery from the inferior wall should not be ignored, and cares should be taken when exploring the occluded segment beyond the lower edge of the ICA contour. When exploring the ophthalmic segment which is bent, the guide wire can easily exert force on the local anterior upper wall and cause dissection. It is recommended to use a microcatheter with the tip shaped to 45°. It may be more reasonable for the guide wire to deviate to the lower wall in the proximal segment and to the upper wall in the distal segment when exploring the occluded segment. If a dissection in the ICA is too long and the micro-guide wrie cannot enter the true cavity in time, it is wise to terminate the operation because repeated operation may damage the opening of the posterior communicating artery and destroy formed collateral compensation.

### Recanalization of MCA occlusion

Recanalization of MCA occlusion is mainly at the M1 segment. Because the blood vessels at the M2 segment and beyond are small, recanalization of these vessels is highly risky and rarely performed^5,28,29^. The course of the occluded M1 segment can refer to that of the other side, with the course being mostly straight towards the out rear side, and the M1 segment measures 15–20 mm in length and 2.0–2.5 mm in diameter. The posterior superior wall sends out slender lenticular arteries while the anterior inferior wall sends out the temporal branches^34^. In our study, the successful recanalization rate for MCA occlusion was 88.5%, with the highest periprocedural complication rate of 17.3% among all patients and a reocclusion rate of 4.3%. There are many perforating arteries in the M1 segment of the MCA, and serious infarction of the conducting fibers in the basal ganglia and radial crown may lead to severe disability and poor prognosis if these perforating arteries are injuried during endovascular operation for recanalization. Moreover, the tip of the micro-guide wire can enter the slender insular arterial branches of the M2 segment and injure them if maneuvered roughly, resulting in intracerebral hemorrhage and a higher periprocedural complication rate. In order to reduce the perioperative complication rate, suboptimal recanalization of the MCA occlusion is often performed with greater residual stenosis, which may lead to poor follow-up outcomes. In patients with better effects of MCA recanalization, the imaging manifestations are mostly watershed infarction and large-scale cortical hypoperfusion, which is caused by short occlusion of M1 segment with the original stenosis locating in the front and lower part of M1 without occluding the perforator artery of the MCA. The potential lacuna and thrombus-occluded segment are located in the rear and upper part of M1 segment, which is right in the direction of exploration of the micro-guide wire.

### Recanalization of VBA occlusion

The intracranial segment of the vertebral artery runs straight, and its branches are mainly the posterior inferior cerebellar artery, anterior spinal artery and some perforating arteries of the medulla oblongata^26^. If occlusion of the intracranial segment involves the posterior inferior cerebellar artery, it can cause a large area of cerebral infarction in the lower part of the cerebellar hemisphere. When the infarction exceeds 1/3 of the cerebellar hemisphere, cerebellar tonsillar hernia may take place. If the anterior spinal artery or perforator artery of medulla oblongata is occluded, respiratory and swallowing functions will be affected, leading to poor consciousness and severe clinical symptoms. The normal basilar artery courses straight and measures on average 3.2 mm in diameter and 2.5 mm–3.0 cm in length. The dorsolateral perforating branches are abundant and supply blood to the midbrain and brainstem. Occlusion of longer segments often leads to serious disability, with little significance for recanalization in non-acute stage. In patients with short occlusion and poor collateral compensation, the symptoms may be repeated with delayed progression, and recanalization may benefit these patients at the non-acute stage^27,31,32^. In our study, the occlusion was located in the intracranial VBA which was recanalized in 82.8% patients with a periprocedural complication rate of 5.2%.

### Calcification, dissection and complications

For patients with eggshell-like and long-segment calcification occlusion on imaging presentation, we should retreat from trying to recanalizing the occlusion. Even if the occlusion is recanalized, severe perioperative complications and poor long-term prognosis may be resulted because calcification is significantly associated with successful recanalization as found in our study. If arterial dissection is mistakenly created, the operation should be terminated immediately to prevent further damage to the vessel, especially in the intracranial segment of vertebral artery. This segment has small blood vessels supplying the medulla oblongata, which is closely related to the respiratory center, and injury to these vessels may lead to acute or subacute respiratory disorders. In many cases of injury, there are no premonitory symptoms, and special care should be taken in clinical postoperative management to prevent apnea or asphyxia. To prevent and reduce periprocedural complications, medical imaging before recanalization should be carefully evaluated for the occlusion length and vessels distal to the occlusion. High-resolution MRI is helpful to distinguish the plaque orientation and true segments of occluion. In some patients, angiography through a micro-catheter proximal to the occlusion can be used to assess the occlusion.

### Clinical outcomes and patient selection

In our study, over 80% of the patients achieved an mRS score of 0 at 90 days post recanalization. The prognoses were indeed very good. Possible reasons were described as follows. Most patients enrolled had only mild symptoms with the mRS ≤ 2 in 91.2% and mRS > 2 in the rest 8.8% (n = 16). Patients with mRS > 2 had definitive hypoperfusion which would result in good prognosis after successful recanalization and restoration of perfusion. Moreover, a considerable number of patients were in the recovery stage, and the effect of natural functional recovery plus endovascular reconstruction of blood flow resulted in good prognosis. Endovascular recanalization and restoration of blood flow improved the hypoperfusion in some brain tissues and restored the function of some brain dormant parts at hypoperfusion. On the other hand, our study suggests that patients with mild disability and insufficient blood perfusion are one of the potential indications for recanalization in the non-acute phase of cerebral artery occlusion. In this study, all enrolled patients had survived the initial acute stage of arterial occlusion. Patients requiring emergency surgery, with severe disability or insignificant hypoperfusion were not included. For patients with short segment of occlusivon and a small amount of compensation in the distal segment, neurological symptoms will likely recur due to insufficient compensation, and for these patients, endovascular recanalization is beneficial. However, the timing of recanalization is difficult to decide. Early recanalization is conducive to saving the brain tissue at risk of poor perfusion and reducing the recurrence and progression of cerebran infarction. Nonetheless, early recanalization may involve fresh thrombus and new infarction, leading to increased risk of perioperative thrombus shedding, bleeding transformation and stent reocclusion^36,37^. Thus, endovascular treatment was selected at around 3 weeks after imaging-confirmed occlusion of intracranial arteries based on studies in the literature^36,37^.

### Factors associated with recanalization, complications and restenosis

Our study identified calcification as one independent factor significantly associated with restenosis or reocclusion. This factor had also been confirmed to significantly link to instent restenosis when using stent angioplasty for intracranial atherosclerotic stenosis^38^. In atherosclerotic diseases, plaque calcification often develops through an inflammation-dependent mechanism of atherosclerotic progression^39^. Moreover, atherosclerotic calcification is frequently coexistent with arterial tortuosity or angulation, whereas angulation is an independent factor significantly affecting successful recanalization and restenosis or reocclusion of in stent recanalization of intracranial arterial occlusion as revealed by our study. Both calcification and arterial angulation result in poor compliance of arteries to balloon angioplasty and necessitate application of a greater balloon inflation pressure^38^, which may cause subsequent arterial dissection or rupture, increasing the periprocedural complication rate. When the calficied or angulated artery of occlusion was not sufficiently dilated, a stent might not be able to apposite closely to the wall, leading to restenosis or reocclusion. Restenosis or reocclusion occurred more often in the ICA ophthalmic segment followed by ICA cavernous segment, VBA, and MCA. The ophthalmic and cavernous segments of the ICA are more curved with thicker arterial wall which are not readily responsive to balloon angioplasty or compliant to stent deployment as the other vessels do. Periprocedural complications like arterial dissection, injury and thrombus formation may cause greater restenosis or reocclusion of stented artery, and with greater follow-up durations, more instent intimal hyperplasia will develop, resulting in more restenosis or reocclusion. Occlusion duration significantly affected successful recanalization because longer time would cause longer and stronger occlusion by intimal hyperplasia. Periprocedural complications occurred more often in recanalization of MCA occlusion than in VBA or ICA as analyzed above, and hyperlipidemia may also significantly affect the complications as revealed by our study. The risk factors for intracranial atehrosclerosis include diabetes mellitus, hypertension, hyperlipidemia and cigarette smoking^40^, however, only hyperlipidemia was identified as an independent significant factor affecting complications, which needs to be confirmed. Moreover, patients’ height was also associated with the presence of complications, which also needs further study for confirmation.

For successful recanalization of ICA and VBA occlusion, angulation and calcification were two independent risk factors. For MCA recanalization, right side occlusion, occlusion length and angulation were three independent risk factors, whereas for VBA recanalization, occlusion length and older age were independent risk factors in addition to angulation and calcification. For restenosis or reocclusion, only mRs at 90 days was an indepent risk factor. For the continuous variable of angulation as a significant independent risk factor for recanalization, the cutoff value was 76.9° for ICA recanalization, 16.0° for MCA recanalization, and 29.4° for VBA recanalization, which indicates that an angulation greater than the above cutoff values will result in an increased successful recanalization rate. The cutoff value of the independe risk factor of occlusion length was 7.3 mm for MCA recanalization and 25.2 mm for VBA recanalization, suggesting an occlusion length greater than the above cutoff values will lead to a decreased recanalization rate.

Based on our outcomes, clinical experience and studies published in the literature, we have coined a risk classification for recanalizing occlusion of large intracranial arteries (Table 5), which needs to be confirmed in clicnial practice.

## Limitations

This study had some limitations, including the retrospective and one-center study, a small cohort of patient, insufficient quantitative perfusion data, patient’s heterogeneity, Chinese patients enrolled only, short-term follow-up, and no randomization, which may all produce some bias to affect the generalization of the study. Explanation of the outcomes should be cautious. Future studies will have to resolve all these issues to improve and confirm the outcome.

## Conclusions

In concsluion, recanalization surgery may be a safe and effective approach for patients with non-acute symptomatic occlusion of large intracranial arteries which do not respond to medications, and factors significantly independently associated with successful recanalization, periprocedural complications and restenosis or reocclusion after recanalization have been identified to improve the outcome in future practice.

## Data Availability

All data related to this article is available from the corresponding author on reasonable requirement.
